# The role of PI3Kα isoform in cardioprotection

**DOI:** 10.1007/s00395-017-0657-7

**Published:** 2017-10-17

**Authors:** Xavier Rossello, Jaime A. Riquelme, Zhenhe He, Stasa Taferner, Bart Vanhaesebroeck, Sean M. Davidson, Derek M. Yellon

**Affiliations:** 1 The Hatter Cardiovascular Institute, University College London, 67 Chenies Mews, London, WC1E 6HX UK; 2 Advanced Center for Chronic Diseases (ACCDiS), Facultad de Ciencias Quimicas y Farmaceuticas and Facultad de Medicina, Universidad de Chile, Santiago, Chile; 3 UCL Cancer Institute, University College London, London, UK

**Keywords:** Cardioprotection, Phosphoinositide 3-kinase, Ischemic preconditioning, Ischemia/reperfusion injury

## Abstract

Ischemic preconditioning (IPC) limits myocardial infarct size through the activation of the PI3K–Akt signal cascade; however, little is known about the roles of individual PI3K isoforms in cardioprotection. We aimed, therefore, to elucidate the role of the PI3Kα isoform in cardioprotection Pharmacological PI3Kα inhibition was assessed in isolated-perfused mouse hearts subjected to ischemia/reperfusion injury (IRI), either during the IPC procedure or at reperfusion. PI3Kα *inhibition* abrogated the IPC-induced protective effect at reperfusion, but not when given only during the IPC protocol. These results were confirmed in an in vivo model. Moreover, pharmacological PI3Kα *activation* by insulin at reperfusion was sufficient to confer cardioprotection against IRI. In addition, PI3Kα was shown to be expressed and activated in mouse cardiomyocytes, mouse cardiac endothelial cells, as well as in mouse and human heart tissue. Furthermore, PI3Kα was shown to mediate its effect though the inhibition of mitochondrial permeability transition pore opening. In conclusion, PI3Kα activity is required during the early reperfusion phase to reduce myocardial infarct size. This suggests that strategies specifically enhancing the α isoform of PI3K at reperfusion promote tissue salvage and as such, and could provide a direct target for clinical treatment of IRI.

**Electronic supplementary material:**

The online version of this article (doi:10.1007/s00395-017-0657-7) contains supplementary material, which is available to authorized users.

## Introduction

Ischemic preconditioning (IPC), whereby brief cycles of non-lethal ischemia and reperfusion protect the myocardium from a subsequent, sustained ischemic insult, was first described by Murry et al. in a canine model in 1986 [35]. Over the last three decades, IPC has been reproduced in all species examined [54], including humans [53], and has also been replicated in other organs when applied either locally or remotely [18]. The underlying mechanisms of IPC have been largely uncovered and subsequently extrapolated to most cardioprotective therapies [13]. IPC has indeed become the paradigm for cardioprotection, although its signalling architecture is still the subject of intense research aimed to identify novel therapeutic targets to treat ischemia/reperfusion injury (IRI).

IPC signal transduction is understood to be mediated by a three-step process [16]: (1) the release of triggers that usually bind sarcolemmal membrane receptors (e.g., bradykinin); (2) the activation of intracellular mediators (signalling cascades that help initiate and propagate the signal, i.e., pro-survival kinases); and (3) the action of an end-effector that attenuates cell injury, i.e., inhibition of mitochondrial permeability transition pore (mPTP).

The IPC-induced protective effect is mediated by pro-survival signalling cascades, such as the Reperfusion Injury Salvage Kinase (RISK) pathway, the Survivor Activator Factor Enhancement (SAFE), and the NO/PKG pathway [9, 16, 26, 29]. The RISK pathway encompasses the activation of two parallel kinases (PI3K and ERK1/2) [14, 54]. PI3K has been demonstrated to play a dominant role within the RISK pathway [12, 40], with insights into its role in mediating IPC protection having been revealed by the use of pan-specific PI3K inhibitors, such as wortmannin and LY294002 [13]. In recent years, isoform-specific PI3K inhibitors have been developed in the field of oncology [51], but these have not yet been explored in the context of myocardial infarction, studies which could aid in the development of PI3K isoform-specific agents thereby further enhancing cardioprotection.

PI3Kα has emerged as a key player in cardiac physiology, improving contractility [30, 52], and promoting physiological exercise-induced growth, but not pathological hypertrophy [33]. Constitutively active PI3Kα has also been demonstrated to improve left ventricular function in a heart failure model [28]. Using broad-spectrum PI3K inhibitors, insulin has proven to be cardioprotective through the activation of PI3K [22]. Based on the notion that insulin has been shown to be a canonical activator of PI3Kα [8, 27], we hypothesized that PI3Kα may play a central role in cardioprotection. We, therefore, studied the importance of PI3Kα either using PI3Kα-selective inhibitors and/or enhancing PI3Kα activity using insulin, or by delaying the opening of its end-effector mPTP through PI3Kα inhibition. The dissection of this signalling pathway could help develop co-adjuvant interventions which, when added to reperfusion therapy, would further protect the heart against myocardial IRI in patients with an acute myocardial infarction.

## Methods

### Animals and chemicals

Animals used were male C57BL/6 mice (9–12 weeks, 24–28 g weight), obtained pathogen free from a single supplier and housed under identical conditions.

BYL719 (Alpelisib) was purchased from Selleck Chemicals. GDC–G326 was obtained from Genentech. Human insulin solution was purchased from Sigma-Aldrich. Dimethyl sulfoxide (DMSO, BDH laboratory supplies, UK) was used as the solvent for BYL719 and GDC–G326 at a final concentration not higher than 0.01%.

Both GDC–G326 and BYL719 are potent and highly selective PI3Kα inhibitors [5, 15, 48]; their dosage being established based on the previous publications [5, 48] and in a dose–response characterization study in this paper (see “Results” section). Insulin was tested as a canonical activator of PI3Kα [8, 27] and used at two doses: 100 mU/mL to characterize PI3Kα inhibitors and 5 mU/mL as a low dose with known cardioprotective properties [1, 23].

### Experimental design and study protocols

Outlines for most study protocols can be found through the figures, as well as in the supplemental material (Supplemental Figures 1–5). Additional protocols were as follows:

1. *PI3Kα protein levels* aimed to characterize and quantify the expression under basal conditions of this PI3K isoform in both primary adult mouse cardiomyocytes and mouse cardiac endothelial cells (MCECs), as well as in mouse and human heart tissue (*n* = 5 per group).
2. *IPC protocol selection* Langendorff-perfused adult mice hearts were randomized into three groups (control, IPC 1 cycle, and IPC 4 cycles) to determine differences in myocardial infarct size and Akt activation.
3. *PI3Kα inhibitor dose selection* aimed to determine the dose of PI3Kα inhibitors against the canonical activator PI3Kα insulin, using Akt as a surrogate for PI3K activation and inhibition in line with published data [21, 31, 32, 50].
4. *PI3Kα inhibition to abolish the IPC-induced cardioprotective effect* (*ex vivo*) aimed to determine the role of PI3Kα as IPC mediator in the ex vivo Langendorff-perfused mouse model, where hearts subjected to 35 min ischemia and 2 h reperfusion [39] received either GDC–G326 or BYL719 during the IPC protocol (which consisted of 4 cycles of 5 min ischemia and 5 min reperfusion), or at reperfusion. Both myocardial infarct size and Akt phosphorylation were assessed separately for each set of experiments.
5. *PI3Kα inhibition to block the IPC-induced cardioprotective effect* (*in vivo*) We used an in vivo mouse model of myocardial infarction (40 min ischemia and 120 min reperfusion) subjected to an IPC protocol, in the presence or absence of the PI3Kα inhibitor GDC–G326, administered through external jugular vein at reperfusion.
6. *PI3Kα activation at reperfusion* (*ex vivo*) aimed to assess whether the administration of the PI3Kα canonical activator (insulin) is sufficient to elicit cardioprotection and whether this effect can be abolished through PI3Kα inhibition. Both myocardial infarct size and Akt phosphorylation were evaluated.
7. *PI3Kα activation in cardiomyocytes and MCECs* aimed to study whether both cell types could potentially be involved in the activation of the PI3Kα pathway (Akt phosphorylation was evaluated).
8. *PI3Kα translational ability* aimed to evaluate whether PI3Kα activation, through its canonical activator in human tissue, can be comparable to the outcome observed in mouse tissue (Akt phosphorylation was evaluated).
9. *Impact of PI3Kα activation on the end-effector mPTP,* aimed to evaluate whether PI3Kα activation delays mPTP opening (hence delaying cell death) using insulin as a canonical PI3Kα activator and the GDC–G326 inhibitor. The outcome was measured as half time to mPTP opening.

### Ex vivo isolated Langendorff-perfused mouse heart model of acute myocardial infarction

Heart isolation and Langendorff perfusion were carried out with filtered modified Krebs–Henseleit buffer (composed of 118 mM NaCl, 25 mM NaHCO_3_, 11 mM glucose, 4.7 mM KCl, 1.22 mM MgSO_4_·7H_2_O, 1.21 mM KH_2_PO_4_, and 1.84 mM CaCl_2_·2H_2_O) aerated with a mixture of O_2_ (95%) and CO_2_ (5%) to uphold pH at 7.40 ± 0.3, as previously described [39]. Briefly, mice were given terminal anesthesia and anticoagulation through an intraperitoneal injection of 60 mg/kg sodium pentobarbitone and 100 IU heparin, respectively. Hearts were then harvested, submerged in ice-cold modified Krebs–Henseleit buffer, and immediately cannulated with a 21-gauge cannula to be retrogradely perfused on a murine Langendorff perfusion apparatus at 80 mm Hg pressure.

Predefined exclusion criteria were as follows: (1) more than 4 min time between heart removal and the start of perfusion in the Langendorff mode; (2) temperature above or below the 37 + 0.5 °C range; and (3) buffer flow rate of the isolated heart of less than 1 mL/min or more than 6.5 mL/min on the Langendorff preparation during the stabilization period. After evaluating for exclusion criteria in an initial 20 min stabilization period, the hearts were subjected to experimental protocols of: (a) perfusion for 30 min to collect heart tissue and study the protein expression of PI3Kα under basal conditions; (b) IRI in the presence of IPC, drug, and/or vehicle, as indicated in each protocol (see Fig. 3a, c; Supplemental Figures 1 and 4); and (c) Akt phosphorylation following a given intervention, such as IPC and/or PI3Kα activation and inhibition (see Fig. 4a; Supplemental Figures 2, 3, and 5).

### In vivo murine model of acute myocardial infarction

C57Bl/6 mice were anaesthetized by intraperitoneal injection of 80 mg/kg pentobarbitone at a concentration of 20 mg/mL in 0.9% (w/v) saline and maintained at 36.5 ± 0.5 °C on a heating mat. Surgery was started after confirming the abolishment of pedal and tail reflexes. Mice were intubated using a 19G cannula and ventilated with room air using a MiniVent, type 845, Small Animal Ventilator (Harvard Apparatus, Kent, UK), at a flow rate of 1.0 l/min with 2 cmH_2_O PEEP, stroke volume 200 μl at 130 strokes/min. All mice were subjected to occlusion of the left anterior descending (LAD) for 40 min, which was verified by ST elevation in the electrocardiogram and by the presence of hypokinesia and pallor in the heart, followed by 2 h reperfusion. After the protocol finished, animals were killed by exsanguination via the thoracic aorta. Afterwards, myocardial IS was measured as described in the next section. A total of 33 mice were used for infarct experiments and were randomly assigned to treatment group. Seven mice died during the procedure and were, therefore, excluded from analyses (three in control group, three in IPC, and one in IPC + GDC–G326); hence, each group included six animals.

Mice were randomized to the following groups: (1) control; (2) IPC; (3) IPC and GDC–G326 at reperfusion; and (4) GDC–G326 at reperfusion (see Fig. 3d for further details). IPC was induced by applying 3 cycles of 5 min ischemia and 5 min reperfusion in the LAD. Vehicle control and IPC mice received 50 μl of 6% DMSO in saline (vehicle). GDC–G326 was dissolved in DMSO and injected via external jugular vein (6 μg per ~ 25 g mouse) at reperfusion in groups 3 and 4.

### Myocardial infarct size analysis

At the end of the protocol, the heart was either removed from the Langendorff rig (ex vivo experiments) or else isolated from the animal and aortic root cannulated (in vivo experiments, and 5 mL of 1% 2,3,5-triphenyltetrazolium chloride (TTC) in phosphate-buffered saline injected through the aortic cannula and incubated for 10 min at 37 °C to demarcate the infarcted (white) vs. viable (red) tissue [25]. For regional IRI experiments, the LAD coronary artery was then re-ligated to perfuse Evans blue dye (2 mL of 0.5%) to delineate the area at risk (AAR).

After the incubation, hearts were frozen overnight at − 20 °C and sectioned perpendicular to the long axis the day after, being the slices transferred into 10% neutral formalin buffer for 1 h at room temperature. Images were taken and coded to blind the analyzer. Planimetry analysis using Image J version 1.47 (NIH, Bethesda, MD, USA) was carried out to quantify myocardial IS as a percentage of the AAR.

### Human atrial tissue acquirement

Human atrial tissues were collected from Barts Heart Centre at St Bartholomew’s Hospital. The study received Local Research Ethics Committee approval (REC No. 00/0275) and was carried out in accordance with the University College London Hospitals NHS Trust guidelines. All patients were provided with a Patient Information Sheet and a verbal explanation of the study, in line with Good Clinical Practice guidelines. All patients provided written informed consent and were free to participate in the Barts Cardiovascular Registry.

All patients were aged 18–80 years and their baseline characteristics were recorded upon consent. Patients with impaired renal or ventricular function, dilated left atria, or a history of arrhythmias or on rhythm stabilising medications were excluded. Patients with arrhythmias were excluded based on a previous publication demonstrating that PI3K activation in atrial appendages from patients with atrial fibrillation was lower compared with tissue from patients in sinus rhythm [37].

Right atrial appendage samples were harvested from patients undergoing cannulation for cardiopulmonary bypass either for coronary artery bypass graft or valve replacement. Once the cardiac surgeon provided the atrial tissue, samples were placed in ice-cold, oxygenated modified Tyrode’s buffer (NaCl 118.5 mM, KCL 4.8 mM, NaHCO_3_ 24.8 mM, KH_2_PO_4_ 1.2 mM, MgSO_4_·7H_2_O 1.44 mM, CaCl_2_·2H_2_O 1.8 mM, glucose 10.0 mM, pyruvic acid 10 mM, pH 7.4) and transferred promptly to The Hatter Cardiovascular Institute at University College London.

Right atrial appendage samples were used for both assessing the basal protein expression of PI3Kα and undertaking a pharmacologic approach with both PI3Kα activator and inhibitor.

### Experimental protocol for adult mouse ventricular cardiomyocyte isolation

Adult mouse ventricular cardiomyocytes were isolated using liberase heart digestion as described previously [38]. Briefly, hearts were excised and cannulated through the aorta before retrograde perfusion on a murine Langendorff apparatus at 37 °C. Following perfusion with buffer (consisted of NaCl 113 mM, KCl 4.7 mM, KH2PO_4_ 0.6 mM, Na_2_HPO_4_ 0.6 mM, MgSO_4_·7H_2_O 1.2 mM, NaHCO_3_ 12 mM, KHCO_3_ 10 mM, Hepes Na salt 0.922 mM, Taurine 30 mM, 2,3-butanedione-monoxime 10 mM and glucose 5.5 mM) for 5 min to clear residual blood, and enzymatic digestion was performed using 30 mL perfusion buffer with 5 mg Liberase (Roche, UK) and 12.5 μmol/L CaCl_2_ for about 20 min. At the end of enzymatic digestion, both ventricles were isolated and mechanically disaggregated in a gentle manner. The resulting cell suspension was filtered through a mesh and transferred for enzymatic inactivation to a tube with 10 mL of stopping buffer (perfusion buffer supplemented with fetal bovine serum 10%), and Ca^2+^ was gradually re-introduced with a three-step increasing CaCl_2_ concentration. Cells were then re-suspended in M199 (Invitrogen, UK) supplemented with l-carnitine (2 mM), creatine (5 mM), taurine (5 mM), penicillin (100 IU/mL), streptomycin (100 IU/mL), and 25 μmol/L blebbistatin.

### Cell culture

Immortalized mouse cardiac endothelial cell (MCEC) is derived from microvascular neonatal mouse cardiac endothelial cells.

MCECs were cultured with Dulbecco’s modified eagle medium (DMEM) supplemented with 10% fetal bovine serum at 80–90% confluence. The cells were kept in an incubator at 37 °C, with 95% O_2_ and 5% CO_2_.

MCECs were used to quantify the protein levels of PI3Kα as well as to study their response to the pharmacological activation and inhibition of PI3Kα.

### Tissue homogenates

Mouse hearts and human atrial tissues were obtained as previously described. Tissue samples were swiftly snap-frozen in liquid nitrogen after being collected and then stored at − 80 °C until further processing. The tissue was homogenized in protein lysis buffer, containing Tris pH 6.8 (100 nM), NaCl (300 mM), NP40 0.5%, Halt protease inhibitor cocktail, Halt phosphatase inhibitor cocktail, and 0.5 M EDTA (all from Thermo Scientific, UK) and adjusted to pH 7.4. Homogenates were then sonicated before being centrifuged at 4 °C to remove the pellet containing debris and DNA.

### Western blot analyses

To ensure equal sample loading, protein concentration was determined using bicinchoninic acid (BCA) protein assay reagent (Sigma, UK) and adjusted accordingly. NuPAGE LDS Sample Buffer (4X) (Thermofisher Scientific, UK) plus 5% β-mercaptoethanol were added to the samples, which were subsequently denatured by heating to 100 °C for 10 min. Samples were then loaded on NuPAGE Novex 10% Bis–Tris protein gels (Thermofisher scientific, UK) using the Mini Protean III system (Bio-Rad, UK). Proteins were transferred onto nitrocellulose blotting membrane (GE Healthcare Life Sciences, UK) through wet transfer in a Bio-Rad Mini Trans-Blot. The membranes were blocked for 1 h using 5% bovine serum albumin/PBS tween and subsequently incubated with appropriate primary antibodies at 4 °C overnight.

The following primary antibodies being used were acquired from Cell Signalling Technology: Akt (#9272), Phospho-Akt (Ser473) (#9271), Phospho-Akt (Thr308) (#2965), and PI3 Kinase p110α (#4249). Anti-GAPDH (mAbcam, #9484) was used as loading control. After overnight incubation, membranes were probed with secondary antibodies. Levels of protein were finally quantified using the Odyssey imaging system from Li-Cor Biosciences (Image Studio Lite Ver 5.2).

### Basal PI3Kα protein expression

Expression of PI3Kα protein levels was evaluated by western blot analyses in mouse heart and human atrial tissues, as well as primary adult ventricular mouse cardiomyocytes and MCECs line.

To quantify protein expression, three different amounts of recombinant PI3Kα protein (1, 3, and 10 ng, PI3Kα from Merck Millipore) were loaded in the gels alongside the samples of interest. After being transferred, membranes were probed with specific primary antibodies for PI3Kα. For each membrane, PI3Kα values were extrapolated using a lineal regression. Results were expressed as ng of PI3Kα per μg of heart protein.

### Basal PI3Kα mRNA expression

Mouse hearts were removed and perfused with PBS to remove the blood and then formalin-fixed, paraffin-embedded, and sectioned.

RNAscope (ACD Bio) was performed according to the manufacturer’s methods. Briefly, slides were deparaffinised for 10 min in Histoclear and then dried in 100% alcohol. Slides were incubated in hydrogen peroxide for 10 min RT, and then washed in distilled water. Antigen retrieval was performed by heating for 15 min at 100 °C in target retrieval reagent, then incubating for 30 min at 40 °C in protease plus. Next, the tissue section was hybridized with the RNAscope^®^ Mm-Pik3ca Probe (catalogue number 313581), for 2 h at 40 °C, washed, and fluorescence developed using the Perkin Elmer TSA plus fluorescein kit. The slides were then counterstained with DAPI to stain nuclei, mounted overnight, and sequentially imaged using the 405 nm (for DAPI) and 488 nm laser lines of a Leica confocal microscope and emission filters of 410–480 and 495–550 nm. Positive control slides using the positive control probes supplied in the kit, and negative controls in the absence of hybridization probe were performed in parallel.

### Pharmacologic activation and inhibition of PI3Kα in cells and tissues

Akt phosphorylation was evaluated by western blot analyses after the pharmacologic activation and inhibition of PI3Kα in tissues (mouse heart and human atrial tissues) and cells (primary adult ventricular mouse cardiomyocytes and MCECs).

Prior to being subjected to the pharmacologic stimulation, MCECs were deprived of serum for 2 h. Both cell types were incubated for 15 min according to the following interventions: (1) vehicle control (0.01% DMSO); (2) Insulin (5 mUl/mL); (3) Insulin 5 mUl/mL with 30 min pretreatment GDC–G326 3 μM; and (4) GDC–G326 3 μM.

Mouse Langendorff-perfused hearts were stabilized for 20 min and then perfused for 15 min with: (1) vehicle control (DMSO) perfusion; (2) Insulin (5 mUl/mL); (3) Insulin 5 mUl/mL and GDC–G326 3 μM; and (4) GDC–G326 3 μM.

After being collected in the theatre, human atrial tissue was dissected in four pieces and swiftly submerged in previously oxygenated Tyrode’s modified buffer (NaCl 118.5 mM, KCL 4.8 mM, NaHCO_3_ 24.8 mM, KH_2_PO_4_ 1.2 mM, MgSO_4_·7H_2_O 1.44 mM, CaCl_2_·2H_2_O 1.8 mM, glucose 10.0 mM, pyruvic acid 10 mM, pH 7.4) for 30 min in the presence of: (1) vehicle control (DMSO); (2) Insulin (5 mUl/mL); (3) Insulin 5 mUl/mL and GDC–G326 3 μM; and (4) GDC–G326 3 μM. Of note, the tissue was not perfused, but superfused and under ongoing hypoxia whilst being transferred to the laboratory.

### mPTP opening assay

The sensitivity of the mPTP to opening was assayed using a well-characterized cellular model of reactive oxygen species (ROS)-mediated mPTP opening [6]. Briefly, tetra-methyl rhodamine methyl ester (TMRM, from S Sigma-Aldrich, UK), a lipophilic cation which is very positively charged, accumulates selectively into the negatively charged mitochondrial matrix. At high concentration (12 μM), TMRM becomes quenched at the mitochondria. Constant confocal laser stimulation (at 543 nm wavelength) of TMRM generates ROS within the mitochondria thereby simulating mitochondrial ROS production during reperfusion. After a few minutes of continual confocal laser scanning, ROS induces mPTP opening, producing a drop in mitochondrial membrane potential and resulting in the dequenching of TMRM, which in turn relocates to the cytoplasm [7]. This leak of the dye from the mitochondria to the cytosol increases the detectable fluorescent signal, which is used as surrogate marker for mPTP opening.

Adult mouse ventricular cardiomyocytes were isolated as previously described. Live cardiomyocytes were incubated with the fluorescent dye TMRM at 12 μM for 15 min in Hepes-based recording buffer (NaCl156 mM, KCl 3 mM, MgSO_4_.7H_2_0 2 mM, K_2_HPO_4_ 1.25 mM, CaCl_2_ 2 mM, HEPES 10 mM, and d-glucose 10 mM; pH 7.4), then washed and randomly treated for 15 min into the following groups: (1) vehicle control; (2) insulin 5 mU/mL; (3) insulin 5 mU/mL with GDC–G326 3 μM; and (4) GDC–G326 3 μM alone. Once washed for a second time, mouse cardiomyocytes were stimulated with laser illumination and imaged using confocal microscopy. The time to reach half peak signal was recorded in seconds and compared across groups. A total of 19 ± 2 cardiomyocytes were analyzed for each intervention in each experiment (*n* = 8 mice).

### Statistical analyses

Normal distribution of each data subset was tested using graphical methods and the Kolmogorov–Smirnov method. All values are presented as mean ± standard error of the mean. Comparisons between protein content of PI3Kα were performed using the unpaired *t* test or the Mann–Whitney test depending on the normal distribution of the data. All other continuous data were compared either using one-way analysis of variance if normally distributed or using the non-parametric Kruskal–Wallis test if highly skewed distributed. *P* values for post hoc pairwise comparisons to the control group were adjusted using the Dunnett’s test if normally distributed or the Dunn’s test if non-normally distributed. A *P* value of less than 0.05 was considered statistically significant. STATA software version 13.1 (Stata Corp, College Station, TX, USA), SPSS Statistics version 21 (IBM, Armonk, NY, USA), and GraphPad Prism version 6.00 (GraphPad Software, La Jolla, CA, USA) were used to perform the analyses and produce the graphs. The results were reported according to the ARRIVE guidelines for reporting animal research [24].

## Results

### PI3Kα expression levels in cardiac tissue and cells

**Fig. 1:**
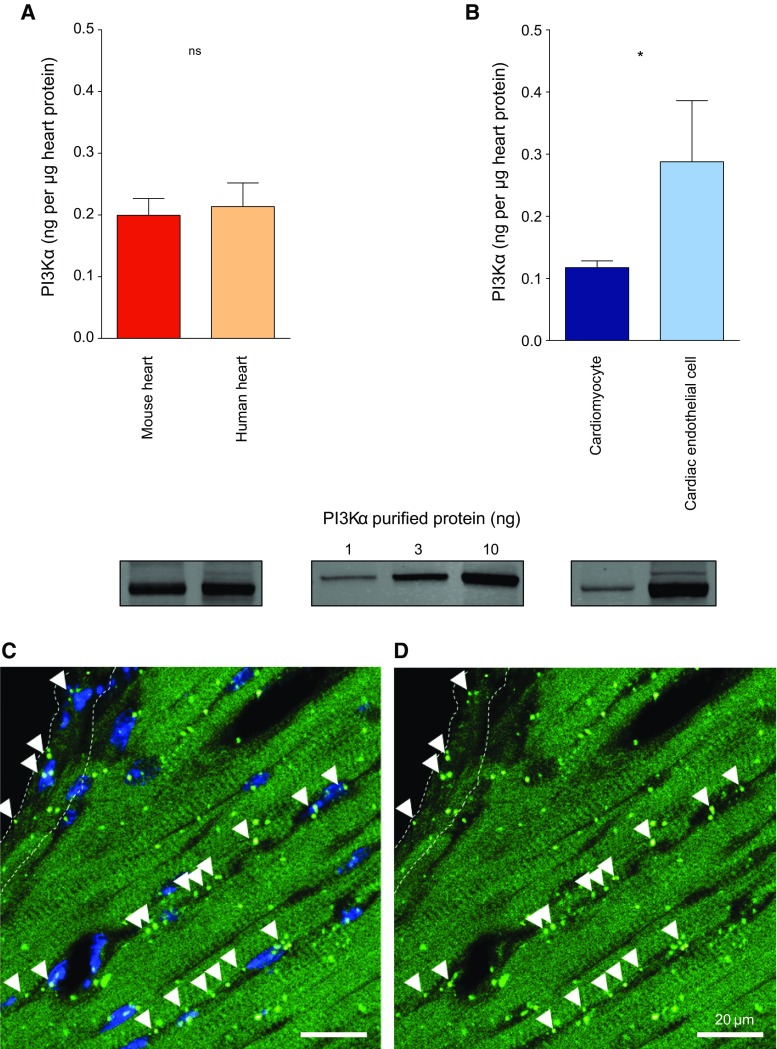
PI3Kα protein and mRNA levels. PI3Kα protein expression in mouse and human cardiac tissue (**a**) and cardiomyocytes and endothelial cells (**b**). PI3Kα values were extrapolated using a lineal regression from three different amounts of recombinant PI3Kα (1, 3, and 10 ng). Results are presented as mean ± SEM and expressed as ng of PI3Kα per μg of heart protein. PI3Kα mRNA expression in mouse cardiac tissue (**c**, **d**, with and without DAPI-stained nuclei in blue, respectively). RNAScope, a high sensitivity method of in situ hybridization, was used to specifically detect PI3Kalpha mRNA expression in mouse heart sections. Bright dots represent PI3Kalpha mRNA. A few dots are visible in the cytosol of cardiomyocytes (visible from their green autofluorescent background), but there are proportionally many more dots in the endothelial cells of capillaries running alongside them, as indicated by arrow heads, and in endothelial cells lining larger vessels (demarcated by dotted lines). Scale bar 20 μm. *P < 0.05, **P < 0.01, ***P < 0.001, and *ns* non-significant

We first quantified PI3Kα protein expression in mouse and human heart tissue relative to recombinant PI3Kα standards (Fig. 1a). In the tissue, PI3Kα was found to be expressed at a similar level of total protein in both mouse and human heart, respectively (0.20 ± 0.03 and 0.21 ± 0.04 ng per μg heart protein, *P* = 0.76). At a cellular level, PI3Kα was 2.5fold more highly expressed in endothelial cells than in cardiomyocytes (Fig. 1b). PI3Kα mRNA expression in mouse cardiac tissue (Fig. 1c, d) also suggests a greater expression of this isoform in endothelial cells compared to cardiomyocytes.

### Selection of IPC protocol and PI3Kα inhibitor dose

**Fig. 2:**
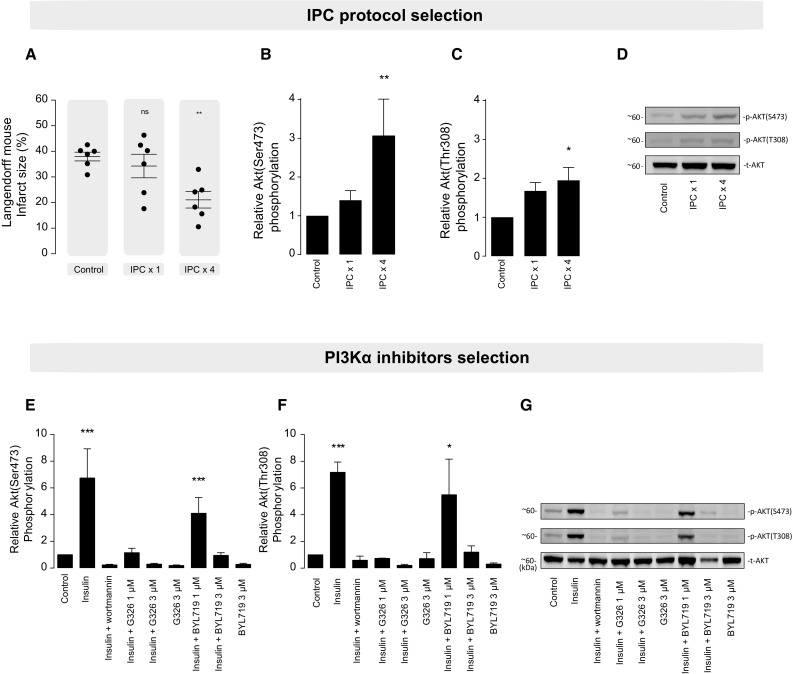
IPC protocol and PI3Kα inhibitors dose selection. **a** Effect of two ischemic preconditioning (IPC) protocols on myocardial infarct size (%). **b**–**d** Impact of IPC protocols on Akt phosphorylation. **e**–**g** Dose–response curve of two PI3Kα inhibitors (BYL719 and GDC–G326) when co-administered with the α canonical activator. *P < 0.05, **P < 0.01, ***P < 0.001, and *ns* non-significant

To select an IPC protocol able to confer protection in an ex vivo model, we compared preconditioning strategies of 1 cycle vs. 4 cycles. IPC 4 cycles demonstrated a significant reduction of myocardial infarct size (IS) compared to control group (21.1 ± 3.2 vs. 38.0 ± 1.7%, *P* = 0.006), whilst IPC 1 cycle was not effective (34.3 ± 4.6, *P* = 0.67 compared to control group) (Fig. 2a). PI3K pathway activation, as assessed by the levels of phospho-serine473 and phospho-threonine308 Akt levels, was increased after IPC 4 cycles compared to the control group, whilst Akt phosphorylation after IPC 1 cycle was not significantly increased (Fig. 2b–d).

To identify appropriate doses of PI3Kα inhibitors, we generated a dose–response curve using GDC–G326 and BYL719, two distinct PI3Kα-selective inhibitors, in the context of stimulation with insulin, a known canonical activator of PI3Kα. These experiments indicated that 3 µM of each drug is sufficient to inhibit Akt(Ser473) and Akt(Thr308) phosphorylation induced by insulin (Fig. 2e–g). Hence, these concentrations were used to evaluate the role of PI3Kα in cardioprotection in subsequent experiments.

### PI3Kα mediates protection by IPC against myocardial IRI

**Fig. 3:**
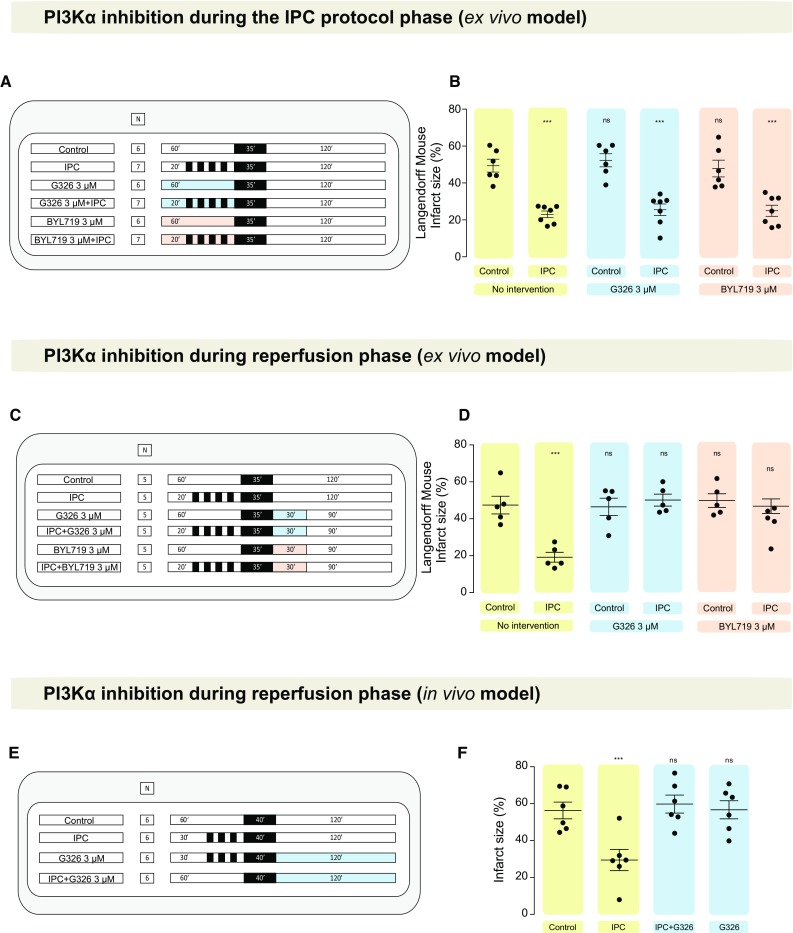
PI3Kα inhibition in ischemic preconditioning. Effect on myocardial infarct size of two PI3Kα inhibitors (BYL719 and GDC–G326) when administered during the IPC protocol (**a**, **b**) or at reperfusion (**c**, **d**). The study protocol and results in the in vivo model are depicted in **e** and **f**, respectively. *P < 0.05, **P < 0.01, ***P < 0.001, and *ns* non-significant. *IPC* ischemic preconditioning

**Fig. 4:**
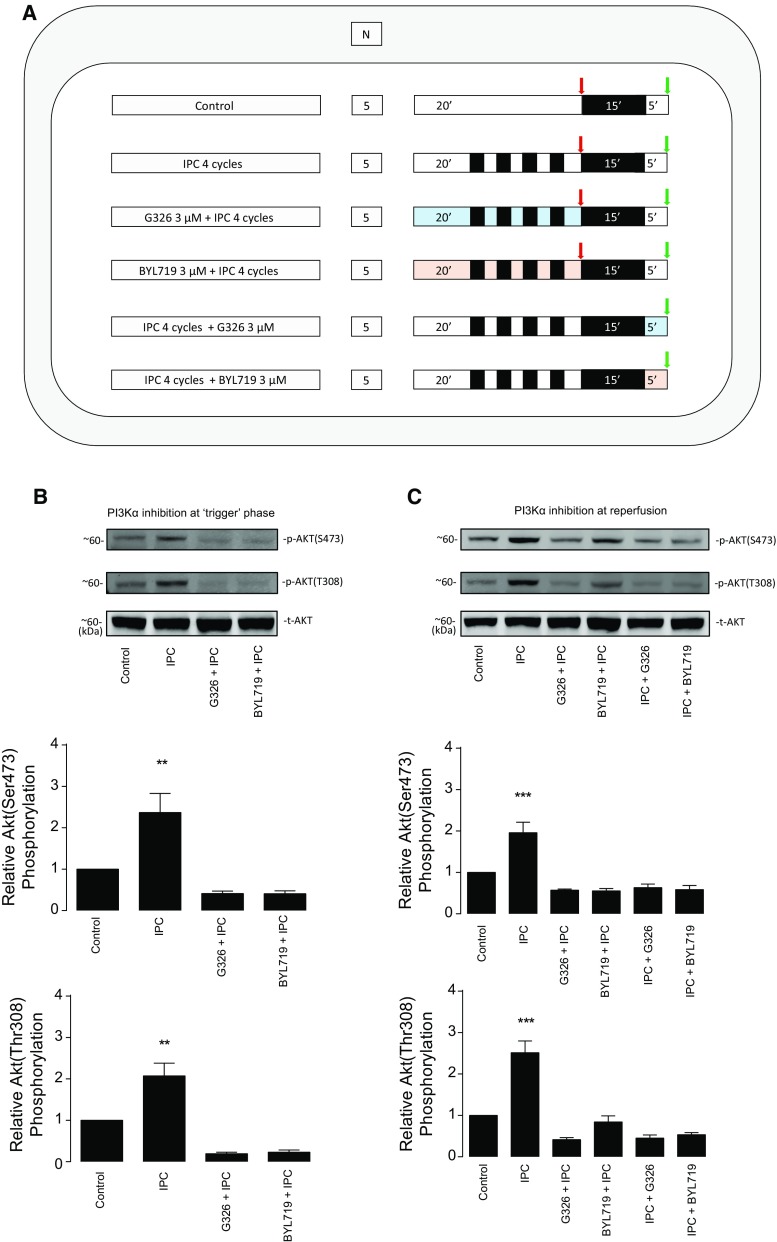
Impact of PI3Kα inhibition on Akt phosphorylation during the IPC protocol and at reperfusion. **a** Overview of protocols performed to assess Akt phosphorylation using western blot analysis. Black boxes represent periods of ischemia, white boxes represent periods of perfusion with Krebs–Henseleit buffer at 80 mmHg, and coloured boxes represent the perfusion of a given drug (turquoise for G326 and salmon for BYL719). Arrows represent the moment, where samples were collected (in red, all samples collected after IPC protocol; in green, all samples collected at reperfusion). **b** Akt activity measured after IPC in the presence or absence of PI3Kα inhibitors (i.e., during the IPC protocol) and **c** Akt activity measured at reperfusion following PI3Kα inhibition during either IPC protocol phase only (“drug + IPC”), or at reperfusion (“IPC + drug”). *IPC* ischemic preconditioning

In the Langendorff-perfused, ex vivo mouse heart model, IPC reduced IS compared to control (49 ± 4 vs. 23 ± 2%, *P* < 0.001). This protection was unaffected by GDC–G326 (26 ± 3%) or BYL719 (25 ± 3%) when administered only during IPC, despite Akt phosphorylation being inhibited (Figs. 3a, b, 4b). Importantly, these two structurally distinct inhibitors did abrogate protection when given at reperfusion (GDC–G326: 50 ± 3%; BYL719: 47 ± 4%) and also blocked Akt phosphorylation (Figs. 3c, d, 4c), revealing a distinct role for the PI3Kα isoform during the early moments of reperfusion. Neither drug affected IS on its own.

These experiments were confirmed in an in vivo setting using GDC–G326, which was found to block IPC protection when administered at reperfusion (control 56 ± 5 vs. IPC + GDC–G326 60 ± 5%, *P* = 0.93, and IPC alone was 29 ± 6%, *P* = 0.003) (Fig. 3e, f).

### Insulin, a canonical activator of PI3Kα, is protective at reperfusion

**Fig. 5:**
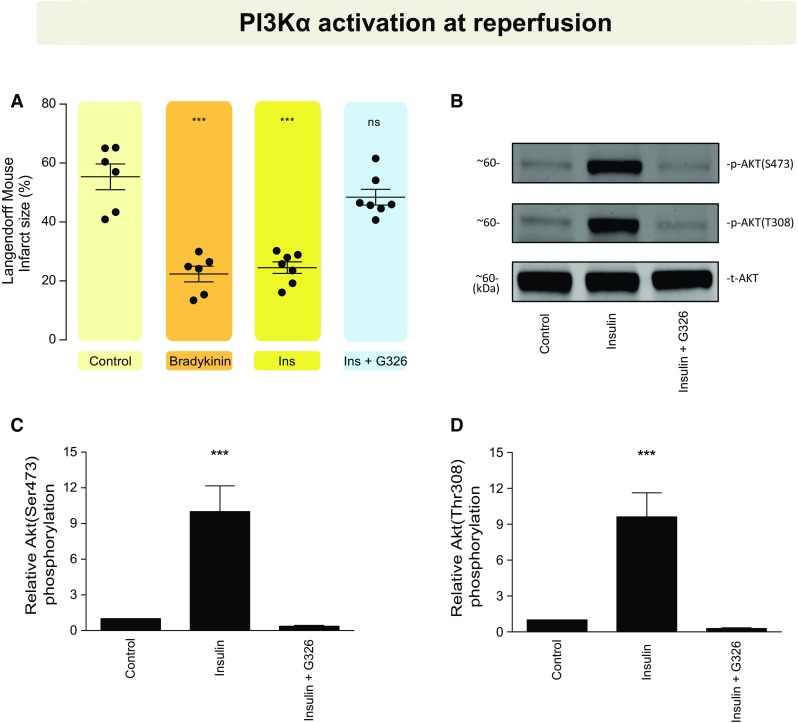
Pharmacological PI3Kα activation at reperfusion. **a** Effect on myocardial infarct size of PI3Kα canonical activator (insulin) administered at reperfusion. **b**–**d** Western blot analyses. *P < 0.05, **P < 0.01, ***P < 0.001, and *ns* non-significant

Having established that PI3Kα is necessary to *mediate* the protection provided by preconditioning, we evaluated whether activation of PI3Kα at reperfusion would be sufficient to elicit a protective effect. When administered at reperfusion, the canonical PI3Kα activator (insulin) reduced myocardial IS compared to control (25 ± 2 vs. 55 ± 4%, *P* < 0.001) and this protection was abolished by GDC–G326 (48 ± 3%, *P* = 0.687 compared to control), as depicted in Fig. 5a. The degree of protection was similar to that attained using bradykinin as a pharmacological positive control. Accordingly, Akt was activated by insulin and blocked when GDC–G326 was co-administered at reperfusion following a protocol of IRI (Fig. 5b–d).

### PI3Kα can be activated in mouse cardiomyocytes, endothelial cells, and atrial human tissue

**Fig. 6:**
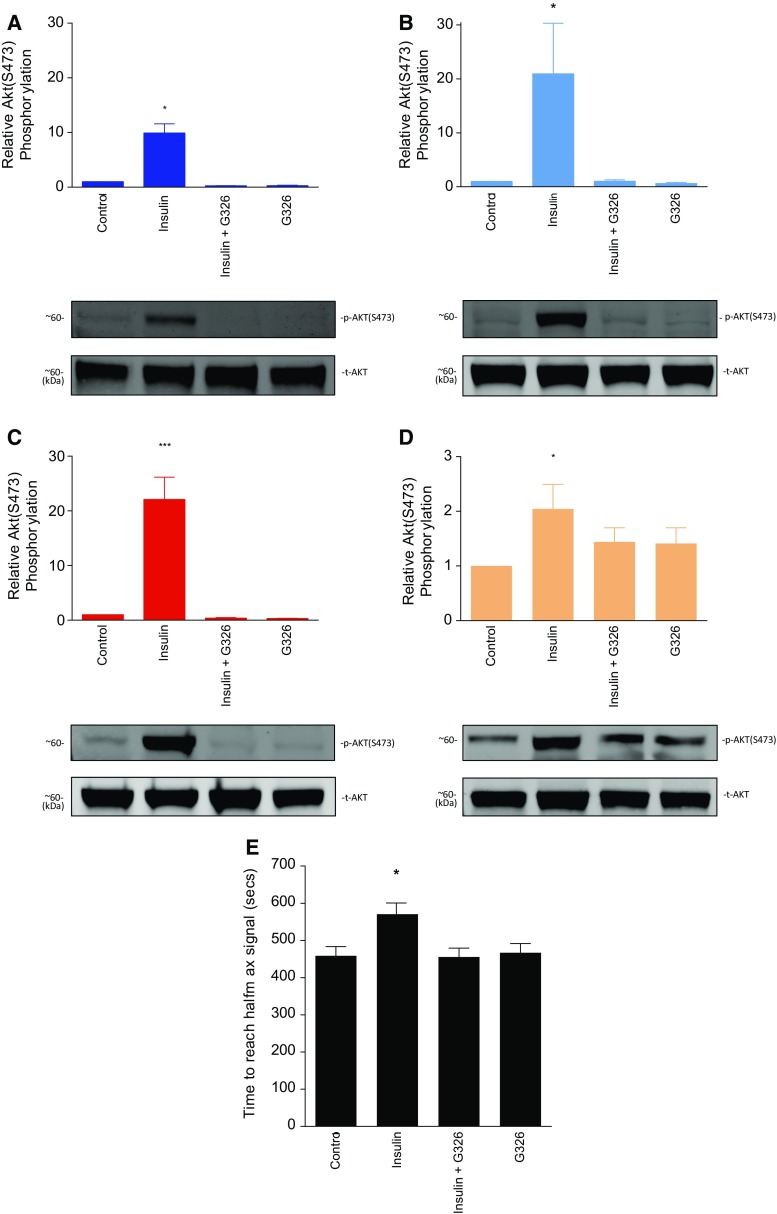
Pharmacological PI3Kα activation in cells and tissues. Akt activation in response to treatment with insulin and the GDC–G326 inhibitor in adult mouse ventricular cardiomyocytes (**a**), mouse cardiac endothelial cells (**b**), isolated-perfused mouse heart tissue (**c**), and human atrial tissue (**d**). **e** ROS-stimulated mPTP opening in cardiomyocytes was de-sensitize by prior insulin treatment. *P < 0.05, **P < 0.01, ***P < 0.001, and *ns* non-significant

Having established a central role of PI3Kα in cardioprotection, we aimed to determine whether insulin stimulated PI3Kα activation only in cardiomyocytes or also cardiac endothelial cells. Our results show that Akt is activated in both cell types in response to insulin and this effect was lost upon co-administration with GDC–G326 (Fig. 6a, b). Furthermore, to assess the translation potential of our findings, we collected human right atrial appendage samples and tested whether the PI3Kα pathway could be stimulated (patient baseline characteristics are outlined in Table 1). Human tissue was shown to respond to insulin with PI3Kα-dependent phosphorylation of Akt, although the fold increase was lower due to a higher basal activity (Fig. 6c, d).

**Table 1:** Patient baseline characteristics

	Total patients (*n* = 5)
Age	64.2 ± 18.6
Gender (male)	4 (80%)
Diabetes mellitus	1 (20%)
Dyslipidemia	2 (40%)
Hypertension	5 (100%)
Smoking history	1 (20%)
Prior cardiovascular disease	2 (40%)
Preserved LVEF (> 50%)	4 (80%)
Sinus rhythm	5 (100%)
Surgery	
CABG	3 (60%)
AVR	1 (20%)
CABG + AVR	1 (20%)
Medications	
Beta blocker	4 (80%)
ACE inhibitor	2 (40%)
Calcium channel blocker	1 (20%)
Statin	4 (80%)
Antiplatelet therapy	4 (80%)

Data expressed as number (%) or mean ± SD

*AVR* aortic valve replacement, *CABG* coronary artery bypass graft, *LVEF* left ventricular ejection fraction

### PI3Kα activation delays the mPTP opening

The sensitivity of the mPTP to opening was assayed in adult mouse ventricular cardiomyocytes using a well-characterized cellular model of ROS-mediated mPTP opening. In the presence of insulin, the time taken to induce mPTP opening (a surrogate for cell death) was significantly increased compared to control (571 ± 30 vs. 459 ± 25, *P* < 0.013), whilst treatment with GDC–G326 abrogated this effect (455 ± 24 s, *P* = 0.999) (Fig. 6e), GDC–G326 having no effect on its own (467 ± 25 s, *P* = 0.993). These results suggest that PI3Kα signalling promotes cardiomyocyte survival through the inhibition of the end-effector, mPTP.

## Discussion

### Summary of findings

Using a targeted pharmacological approach, we show that PI3Kα activity is required during the early reperfusion phase to reduce myocardial infarct size, as demonstrated with IPC in both ex vivo and in vivo myocardial infarction models. In addition, the exogenous administration of insulin, a canonical activator PI3Kα, was also shown to confer cardioprotection, further demonstrating the importance of PI3Kα in this setting. The potential translational ability of PI3Kα was demonstrated by showing it to be similarly expressed in both human and mouse heart tissues and to respond to insulin in both tissues. At a cellular level, PI3Kα expression was shown to be higher in mouse cardiac endothelial cells compared to cardiomyocytes, with its effect being mediated through the inhibition of mPTP opening.

### Role of PI3Kα and other isoforms in IPC

The RISK pathway has been widely demonstrated to be involved in the protection against IRI conferred by both mechanical and pharmacological conditioning strategies carried out at reperfusion [13, 14, 54]. PI3K is a component of this signalling cascade and, therefore, is an important therapeutic target. Nonetheless, little is known about the role of the distinct PI3K isoforms in IRI setting. Using a pharmacological approach, we have demonstrated that PI3Kα mediates the IPC-induced protective effect during the early moments of reperfusion, but appears not to have a role during the application of the IPC protocol. This is surprising as Akt phosphorylation was abolished at both timepoints following PI3Kα inhibition. Assuming that the specific-PI3Kα inhibitors do not block other isoforms, it might be speculated that PI3Kα-induced Akt phosphorylation is not relevant to promote myocardial salvage during the IPC protocol. The increase of PIP3 following PI3K activation by IPC would not only translate in Akt phosphorylation (which might be irrelevant in this phase) [36], but in other unrevealed cellular mechanisms.

Consistent with our results, transgenic experiments have indicated that PI3Kα improves left ventricular function in a mouse myocardial infarction-induced heart failure model [28], as well as improving contractile dysfunction [30, 31] and preventing exercise-induced cardiomyopathies [32].

In the heart, the most highly expressed PI3K isoforms are PI3Kα and PI3Kβ [10], although little is known about their absolute and relative amounts. PI3Kα can be exclusively activated by tyrosine kinase receptor, whilst there is compelling evidence demonstrating that both tyrosine kinase receptor and G protein-coupled receptors (GPCR) can engage PI3Kβ [4, 11], which is in turn involved in Akt activation in platelets, and in thrombus formation and maintenance, but without known evidence on this involvement in IRI and cardioprotection. PI3Kγ signals downstream of GPCRs. As IPC is mostly mediated by GPCRs ligands, this PI3K isoform has raised interest in a cardiac context. Although not examined in this paper, PI3Kγ has been postulated to be a mediator of the protection afforded by IPC, as IPC-induced protection is lost both in transgenic mice with cardiac-specific overexpression of a catalytically inactive mutant PI3Kγ [49] and knockout mice (PI3Kγ^−/−^) [2]. In contrast to genetic approaches, pharmacological strategies are able to provide novel information by permitting temporally restricted inhibition during either the application of the IPC protocol or at reperfusion. Moreover, it is not always clear whether the molecular signalling alterations of genetically modified animal models can be directly attributed to the targeted gene or to the activation of compensatory signalling pathways. Taken together, we might speculate that PI3Kγ, which involves GPCR-ligand activation, may be involved in the protection elicited by IPC during the IPC protocol, whilst PI3Kα, which involves tyrosine kinase activation, is required at reperfusion for IPC to protect against IRI.

### Pharmacological PI3Kα activation at reperfusion

In our study, we have used insulin as the PI3Kα canonical activator [8, 27]. There is a wealth of evidence demonstrating that insulin mimics the IPC stimulus by activating the RISK pathway, therefore, conferring protection against IRI both in vitro and in vivo through the PI3K–Akt kinase cascade [22, 23, 46]. In the swine model, insulin postconditioning has recently been shown to reduce myocardial IS in a dose-dependent manner [46]. Not surprisingly, we found insulin to be protective in our models, which we confirm to specifically be mediated via PI3Kα. Under the hypothesis of protecting the heart from energy depletion [47], insulin has already been tested as a cardioprototective therapy in the clinical setting, with overall disappointing results [34], although in the subgroup of patients presenting with STEMI in the Immediate Myocardial Metabolic Enhancement During Initial Assessment and Treatment in Emergency Care (IMMEDIATE) trial, the administration of glucose–insulin–potassium therapy significantly reduced cardiac magnetic resonance-evaluated infarct size [42]. The fact that we have identified PI3Kα to mediate the insulin-induced protective effect can move the focus from the use of insulin (a “dirty” drug with many side effects, such as hypoglycemia, hypokalemia, and catecholamine elevation) to the development of pharmacological agents specifically targeting PI3Kα. We are not proposing insulin to come to the fore again, but to take advantage of the overwhelming evidence demonstrating its protective effect to further improve our ability to target its downstream signalling.

With regard to the end-effector mechanism mediating the PI3Kα effect, we used the ROS-mediated mPTP model to confirm the involvement of the mitochondria in the insulin-induced PI3Kα activation. Using broad-spectrum PI3K inhibitors, our group had previously linked the activation of the PI3K–Akt pro-survival kinase pathway through insulin with the inhibition of the mPTP [7]. Our pharmacological approach used in cells further suggests that the effect of insulin on the mPTP opening is mediated by PI3Kα, although we lack specific data linking PI3Kα and mPTP in preconditioned cardiomyocytes.

### PI3Kα expression and activation in cells and tissues

PI3Kα is expressed in a similar proportion in both mouse heart and human atrial tissue. Moreover, PI3Kα activity can be pharmacologically modulated in both tissues, as demonstrated using its canonical activator and specific inhibitor. These observations set PI3Kα as a potential target with translational value in cardioprotection. To our knowledge, this is the first study reporting comparisons in PI3Kα protein levels between mouse and human heart tissue. Differences in protein levels between cardiomyocytes and mouse cardiac endothelial cells have also been reported, although caution should be taken when comparing primary isolated with immortalized cultured cells. Our qualitative results on PI3Kα mRNA expression in mouse heart tissue also suggest a greater expression of the isoform in endothelial cells compared to cardiomyocytes in culture. Although cardiomyocytes have become central to recapitulate reductionist models of preconditioning against IRI through hypoxia/reoxygenation experiments, it remains largely unknown to what extent other cardiac cells can contribute in the conditioning phenomena on top of cardiomyocytes. Some advocate that the endothelium might have a relevant role in cardioprotection due to both its optimal situation to interact with blood signals and its paracrine capacity/ability, i.e., nitric oxide has been long associated with ischaemic conditioning through the role of eNOS (the endothelial isoform of nitric oxide synthase) [3]. Besides being a provider of protective triggers and mediators to cardiomyocytes, there is also the possibility for the endothelium to be a target itself for cardioprotection, i.e., preserving microvascular function. Unfortunately, there is little evidence of the specific role of PI3Kα to provide cardioprotection in non-cardiomyocyte cells and this issue needs to be adequately addressed in further studies.

### Translational outlook

The translational perspective of our results is highlighted by the observation that PI3Kα is expressed in human heart tissue and can be stimulated by its canonical activator insulin, although we did not performed experiments addressing the cardioprotective effect of insulin in the human tissue. Given that PI3Kα is involved in the cardioprotection rendered by IPC and insulin at reperfusion, future therapeutic strategies could more specifically target this α isoform of PI3K to enhance its IS-limiting effect in an acute post-myocardial infarction setting, and avoiding the PI3K-independent effects on the metabolism that limits the use of insulin. Thus, further studies with specific PI3Kα activators should be tested in both small- and large-animal models, before being eventually translated in STEMI patients who undergo coronary revascularization. These studies should take into account the current challenges to translate cardioprotective therapies [17, 20, 41].

### Strengths and limitations

To study the activation of the PI3Kα isoform in IPC, we used a pharmacological approach in several experimental models (ex vivo, in vivo, and mPTP assay). Although it could be argued that genetically modified animal models could also have been used to further examine the role of PI3Kα, this would not have been possible due to the fact that chronic deletion of the protein would not allow us to focus on specific phases of the conditioning process, i.e., before ischemia and at reperfusion. To overcome the risk of off-target effects with inhibitors, we used two structurally unrelated inhibitors and observed the same results.

Human heart tissue was obtained from the right atrium. Despite carefully selecting patients without arrhythmias, we appreciate that left ventricular tissue would better represent the human model, but this was not possible to obtain. Moreover, only an insulin-dependent activation of PI3Kα was shown in the human tissue, leaving unanswered the question on whether PI3Kα mediates the IPC-protective effect. Caution should also be taken when extrapolating the results on an immortalized cell line (mouse cardiac endothelial cells) to a more physiological setting.

Several considerations regarding the RISK pathway need to be taken into account regarding the translational value of our results. First, most of the experimental studies involving the RISK pathway as a cardioprotective pathway have been performed in small rodent models of IRI, whereas its central role in large animals is less well established [9, 44]. Second, we focused on classic IPC, not remote preconditioning. Whilst the link between IPC and RISK activation has been demonstrated in humans [43], there is less certainty about the role of the RISK pathway in remote preconditioning in both large-animal models [45] and humans [19].

## Conclusions

PI3Kα activity is required during the early reperfusion phase to reduce myocardial infarct size. The development of drugs enhancing PI3Kα activity at reperfusion could potentially promote myocardial salvage in patients undergoing acute myocardial infarction.

## Electronic supplementary material

Below is the link to the electronic supplementary material.

Supplementary material 1 (DOCX 115 kb)

## Abbreviations

GPCR: G protein-coupled receptor
IPC: Ischemic preconditioning
IRI: Ischemia/reperfusion injury
IS: Infarct size
MCEC: Mouse cardiac endothelial cell
mPTP: Mitochondrial permeability transition pore
RISK: Reperfusion injury salvage kinase
ROS: Reactive oxygen species
