# Supplementary material for: The role of PI3Kα isoform in cardioprotection
Source: Basic Res Cardiol. 2017 Oct 17;112(6):66. doi: 10.1007/s00395-017-0657-7 (PMC5645445; doi:10.1007/s00395-017-0657-7)
Supplement: Supplementary file 1 — Supplementary material 1 (DOCX 115 kb) [file 395_2017_657_MOESM1_ESM.docx]

## SUPPLEMENTAL MATERIAL

#### **Supplemental Fig 1 Study design for the IPC protocol selection**


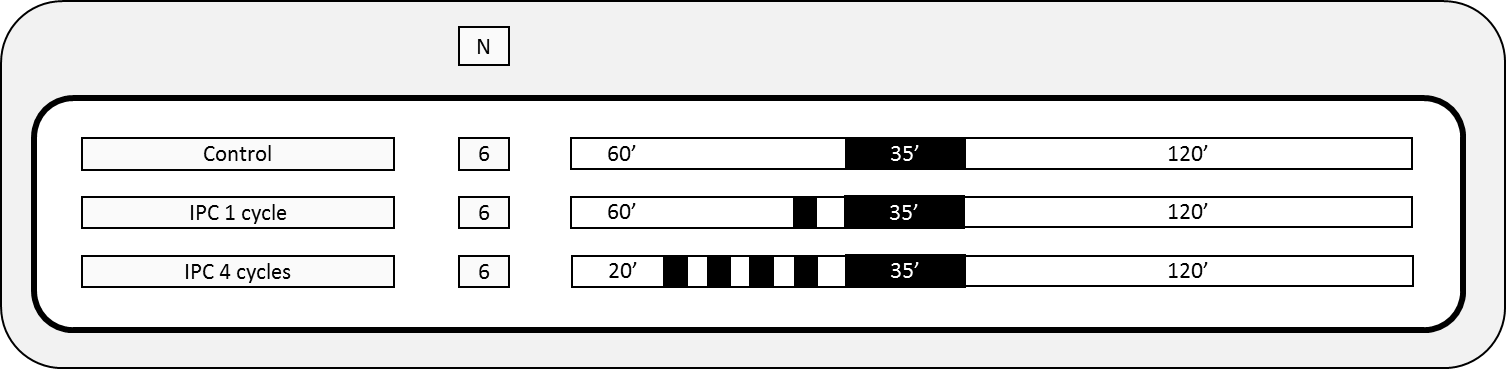


Overview of the Langendorff-perfused mouse protocols aimed to select a protocol for subsequent experiments based on infarct size reduction. A black box represents a period of ischemia and a white box represents a period of perfusion with modified Krebs-Henseleit buffer at 80 mm Hg.

Following 20 min stabilization, three different experimental protocols were tested: (1) control; (2) IPC 1 cycle of 5 min ischemia and 5 min reperfusion; and (3) IPC 4 cycles of 5 min ischemia and 5 min reperfusion per cycle.

IPC indicates ischemic preconditioning.

#### **Supplemental Fig 2 Study design to collect tissue for Western blot analysis to evaluate Akt activation following two separate IPC protocols**


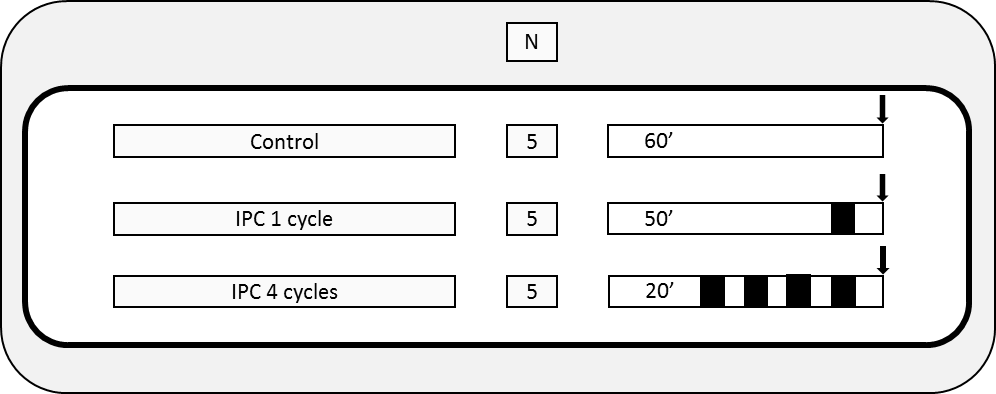


Overview of protocols performed to assess Akt phosphorylation using Western blot analysis. Black boxes represent periods of ischemia, white boxes represent periods of perfusion with Krebs-Henseleit buffer at 80 mm Hg. Arrows represent the moment where samples were collected.

Akt phosphorylation levels was studied in three separate groups: (1) control; (2) IPC 1 cycle of 5 min ischemia and 5 min reperfusion; and (3) IPC 4 cycles of 5 min ischemia and 5 min reperfusion per cycle.

IPC indicates ischemic preconditioning.

#### **Supplemental Fig 3 Study design to collect tissue for Western blot analysis to assess Akt activation to select the adequate use of PI3Kα inhibitors**


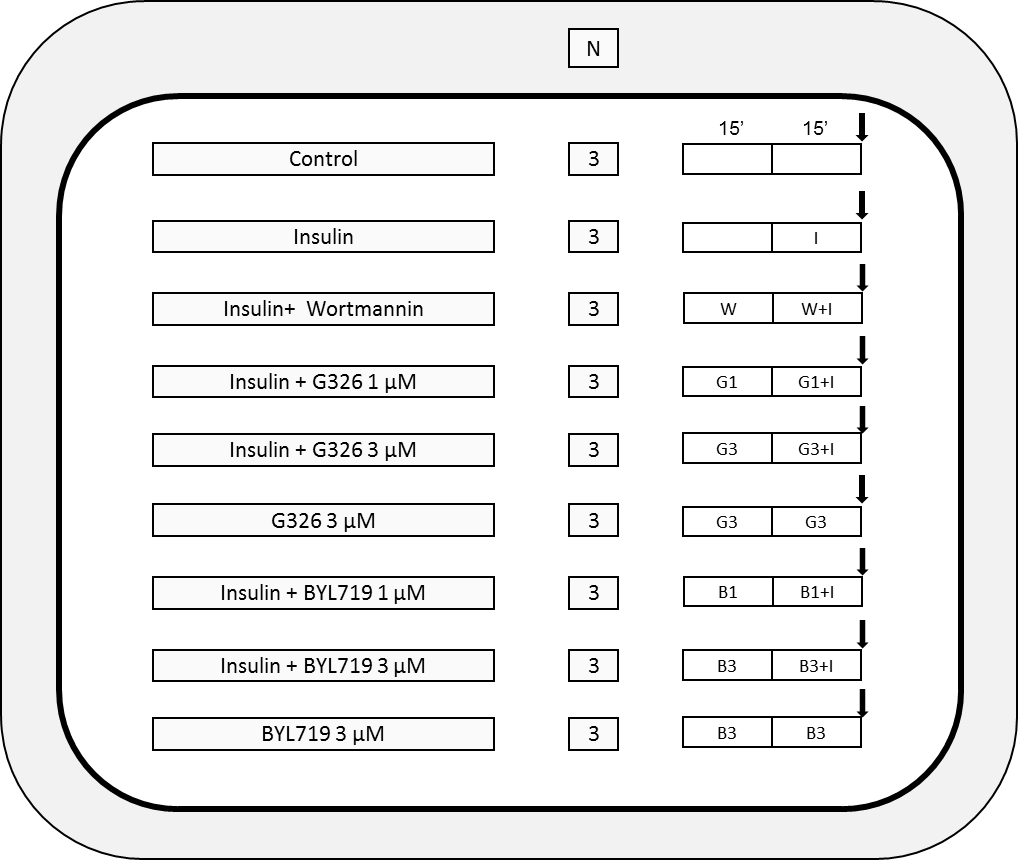


Overview of protocols performed to assess Akt phosphorylation using Western blot analysis. White empty boxes represent periods of perfusion with modified Krebs-Henseleit buffer at 80 mm Hg, whilst white written boxes represent periods of perfusion with the same buffer and the corresponding drug. No protocol of ischemia/reperfusion injury was applied. Arrows represent the moment where samples were collected.

Akt phosphorylation levels were systematically studied in nine separate groups: (1) vehicle control vehicle; (2) insulin 100 mU/mL; (3) insulin 100 mU/mL in hearts pre-treated with wortmannin 100 nM; (4) insulin 100 mU/mL in in hearts pre-treated with GDC-G326 1 μM; (5) insulin 100 mU/mL in in hearts pre-treated with GDC-G326 3 μM; (6) BYL7193μM without insulin; (7) insulin 100 mU/mL in in hearts pre-treated with BYL719 1 μM; (8) insulin 100 mU/mL in in hearts pre-treated with GDC-G326 3 μM; and (9) BYL719 3μM without insulin.

A sample size of 3 animals/group was pre-defined considering both the exploratory purpose of the experiment and the principles of the 3Rs (Replacement, Reduction and Refinement) for humane animal research.

#### **Supplemental Fig 4 Study design to assess the impact on infarct size of PI3Kα activation at reperfusion**


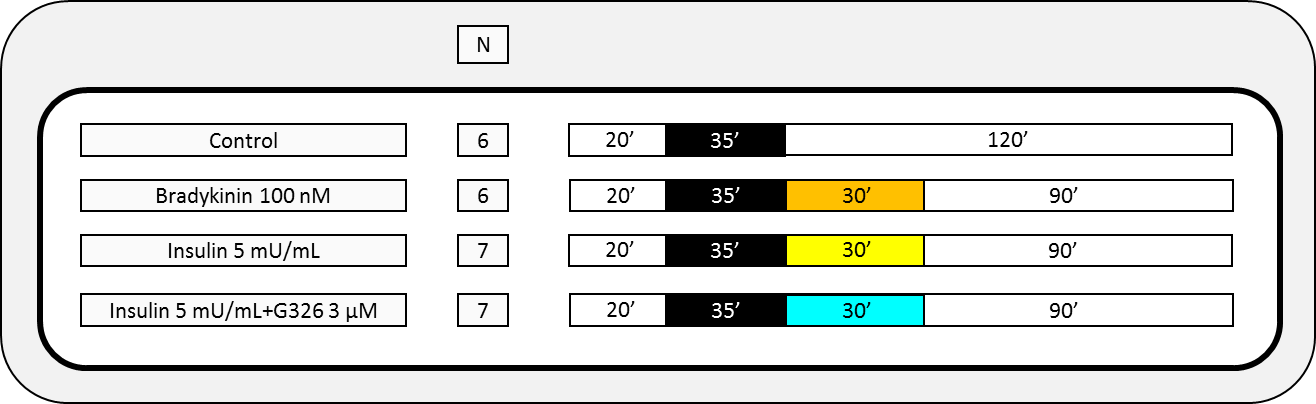


Overview of the Langendorff-perfused mouse protocols aimed to determine the effect on infarct size of PI3Kα activation at reperfusion. A black box represents a period of ischemia and a white box represents a period of perfusion with modified Krebs-Henseleit buffer at 80 mm Hg.

Following 20 min stabilization, four different experimental protocols were tested: 1) vehicle control; 2) bradykinin (well accepted pharmacological positive control) for 30 min; 3) insulin (PI3Kα canonical activator) at a 5 mU/mL concentration for 30 min; and 4) insulin 5 mU/mL and GDC-G326 3 μM co-administered upon reperfusion for 30 min.

IPC indicates ischemic preconditioning. GDC-G326 is a specific-PI3Kα inhibitors.

#### **Supplemental Figures 5 Study design to assess the impact on Akt phosphorylation of PI3Kα activation at reperfusion**


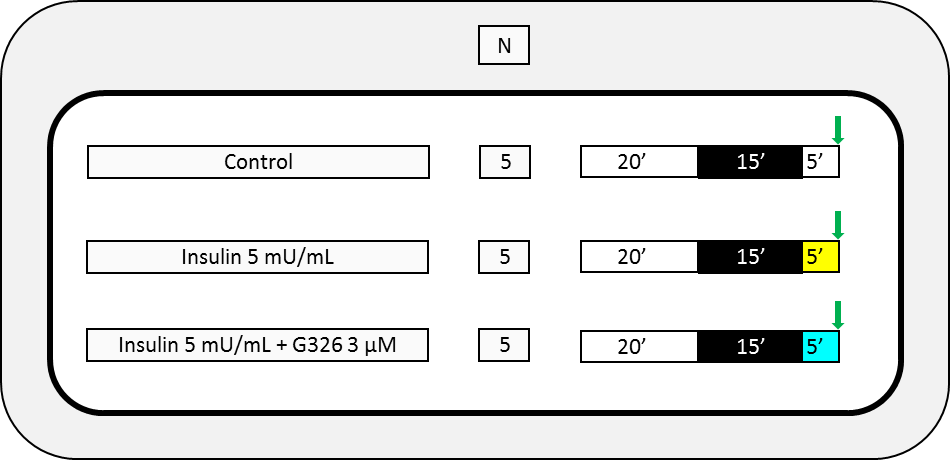


Overview of protocols performed to assess Akt phosphorylation using Western blot analysis. Black boxes represent periods of ischemia, white boxes represent periods of perfusion with Krebs-Henseleit buffer at 80 mm Hg and coloured boxes represent the perfusion of a given drug (yellow for insulin and turquoise for the co-administration of insulin and GDC-G326). Arrows represent the moment where samples were collected (all at reperfusion).

Mouse hearts were randomized into 3 groups in order to assess Akt phosphorylation levels: (1) vehicle control; (2) insulin 5 mU/mL upon reperfusion; (3) insulin 5 mU/mL and GDC-G326 3 μM at reperfusion.
